# Borderline personality disorder (BPD) and attention deficit hyperactivity disorder (ADHD) revisited – a review-update on common grounds and subtle distinctions

**DOI:** 10.1186/s40479-021-00162-w

**Published:** 2021-07-06

**Authors:** Ismene Ditrich, Alexandra Philipsen, Swantje Matthies

**Affiliations:** 1 Department of Psychiatry and Psychotherapy, Medical Center -Faculty of Medicine, University of Freiburg, Hauptstr. 5, D-79104 Freiburg, Germany; 2 Department of Psychiatry and Psychotherapy, Medical Center, University of Bonn, Bonn, Germany

**Keywords:** ADHD, BPD, Impulsivity, Emotion regulation, Comorbidity

## Abstract

**Background:**

Overlap in symptom domains particularly in the field of impulsivity and emotional dysregulation in attention deficit hyperactivity disorder (ADHD) and borderline personality disorder (BPD) have stimulated further research activities since our last review from 2014.

**Main body:**

Disentangling features of impulsivity in ADHD and BPD revealed that impulsivity is a feature of both disorders with patients suffering from both ADHD and BPD having highest impulsivity ratings. BPD individuals have more problems using context cues for inhibiting responses and their impulsivity is stress-dependent, whereas ADHD patients have more motor impulsivity and therefore difficulties interrupting ongoing responses. For emotion regulation difficulties the ranking order ranges from ADHD to BPD to the comorbid condition, again with the patients suffering from both, ADHD and BPD, having the most pronounced emotion regulation problems.

Environmental influences namely adverse childhood events were shown to be linked to both ADHD and BPD. Traumatic experiences seem independently linked to impulsivity features. Thus, some authors point to the risk of misdiagnosis during childhood and the necessity to screen for traumatic experiences in both patient groups.

Genetic research confirmed genetic overlap of BPD with bipolar disorder (BD) and schizophrenic disorders, as well as genetic overlap of BD and ADHD. A population-based study confirmed the high co-occurrence and familial co-aggregation of ADHD and BPD. Interesting questions in the field of gene-environment-interactions are currently dealt with by genetic and epigenetic research.

Few studies have investigated treatment strategies for the comorbid condition, though the issue is highly important for the management of patients suffering from both disorders and presenting with the highest symptom scores.

**Conclusion:**

Research on the different impulsivity features might point to a necessity of disorder-specific treatment strategies in the field of impulse control. Future research is needed to base treatment decisions for the comorbid condition on an evidence basis.

## Background

Attention deficit hyperactivity disorder (ADHD) and borderline personality disorder (BPD) are common psychiatric disorders with prevalences of about 5% for ADHD) [1] and about 1–2% for BPD [2]. BPD is classified as a personality disorder. The DSM-V defines the main features of BPD as a “pattern of instability in interpersonal relationships, self-image, and affects, and marked impulsivity”. ADHD according to the DSM-V is a neurodevelopmental disorder characterized by “a persistent pattern of inattention and/or hyperactivity-impulsivity that interferes with functioning or development” [3]. A high prevalence of ADHD in BPD patients of 30 to 60% was found using structured interviews and self-report questionnaires in retrospective designs [4–6].

ADHD and BPD share important symptom domains, namely impulsivity and emotion dysregulation. Impulsivity as a feature of actions out of spontaneous impulses without thinking about consequences is a core symptom in both disorders, although the psychopathological descriptions of impulsivity in ADHD and BPD in the DSM-criteria are not the same: the type of impulsivity used to define BPD refers to impulsive self-harm and can occur as an associated feature in ADHD, whereas core impulsivity in ADHD is defined as impatience when waiting, talking over people, and interrupting others [3]. Emotion dysregulation is a core symptom of BPD [7] and a frequently occurring symptom in ADHD [8]. It is not part of the official diagnostic criteria for ADHD, as noted in DSM-V, but part of the Wender-Utah-criteria for adults with ADHD [9]. Attention deficit is a core symptom of ADHD and has to date not been considered to be a symptom of BPD, despite in comorbid patients with ADHD and BPD. Shared and overlapping symptoms sometimes lead to difficulties in deciding whether a symptom is part of ADHD or BPD. An interesting scientific debate about the nature of the relationship between the two disorders has stimulated continuing research activities in this field.

Particularly comorbid patients, suffering from ADHD and BPD and their characteristics have been focus of new studies published since 2015 [10–14]. The comorbidity of ADHD, BPD and bipolar disorder (BD) has also been subject of some publications in the field [15, 16]. On a more symptom-oriented base, emotion regulation and impulsivity in different forms and their neurobiological correlates are subject of some mechanism-based and neuropsychological studies since 2015 [11, 12, 17–21]. Discussion is continuing on whether ADHD and BPD occasionally co-occur as comorbidities, have common origins, share common pathological mechanisms, have an additive effect on each other, and/or if ADHD in childhood is a risk factor for the development of later BPD.

This article gives an overview on the state of knowledge on ADHD and BPD and the interrelation between the two disorders updating our review from 2014 on the subject [22].

The aim of this update is to improve and broaden knowledge on the relationship of ADHD and BPD. Information for clinicians will be extracted and a scientifically oriented summary of new findings will be presented to identify research directions.

## Main text

### Method

A search for studies published since 2014 and dealing with ADHD and BPD in adults was conducted for the following bibliographic databases: Pubmed, Embase, Medline, PsychInfo, Central (The Cochrane Central Register of Controlled Trials). The following terms were used: (ADHD OR (attention deficit) OR (attention-deficit) OR hyperactivity*) AND (BPD OR (borderline personality disorder) OR borderline*). The search included all fields in Pubmed, Embase, Medline and PsychInfo databases and abstract, title and keywords in the Central bibliography (Hits: Pubmed 221, Embase 143, Medline 900, Psychinfo 134, Central (The Cochrane Central Register of Controlled Trials) 37).

We searched relevant registers for studies on ADHD and BPD. Furthermore, we screened the reference lists of relevant articles manually. Studies of any design focusing on ADHD and BPD were screened and categorized. We used the categories genetic, epigenetic and environmental risk factors, shared or overlapping symptoms and treatment to group the findings. The systematic search was conducted in April 2020. Originally, as this narrative review is an update to our article from 2014 [22], only studies published between 2014 and 2020 were included. In the course of the peer review process, selected articles were included that were published before this period if they helped to clarify the context of the recent research or after this period if they added interesting data.

### Shared or overlapping symptoms of ADHD and BPD

The main symptomatic overlap between ADHD and BPD is noted in the domains of impulsivity and emotional dysregulation. But on closer examination, these domains are not the same in ADHD and BPD.

#### Impulsivity

Impulsivity is a core symptom of both ADHD and BPD. Impulsivity as a construct remains poorly defined. In a broader sense, „to act without reflecting the consequences" has been considered an acceptable clinical definition of impulsivity. As mentioned above, the descriptive definitions of impulsive behavior used to establish the diagnoses ADHD or BPD are not the same according to the DSM-criteria: the type of impulsivity used to define BPD refers to impulsive self-harm, whereas core impulsivity in ADHD is defined as impatience when waiting, talking over people, and interrupting others from what they are doing [3]. Methods to measure impulsivity differ widely. Psychometric and neuropsychological measures are applied. Frequently used, standardized and validated, is the Barratt Impulsiveness Scale (BIS) as a self-rating instrument for trait impulsivity. The BIS has three subscales for motor impulsivity (acting without premeditating consequences), attentional or cognitive impulsivity (rapid information processing, responsiveness and decisiveness leading to inaccurate actions) and non-planning impulsivity (present oriented inability to prearrange and plan). Neuropsychological measures of impulsivity, e.g. measures of motor impulsivity and cognitive impulsivity, often focus on the behavioral component of impulsivity, namely impulse control or inhibition of stimulus-driven reactions. A classical paradigm to measure motor impulsivity is a go/no go paradigm. Cognitive impulsivity is often measured with delay discounting paradigms where immediate consequences or rewards need to be weighed against future consequences or rewards.

Research suggests that impulsivity is a multifaceted construct that via different traits can lead to maladaptive behavior [23]. In both, ADHD and BPD, impulsivity is a characteristic psychopathological feature, a symptom and diagnostic criterion. Still open remain the research questions if impulsivity in BPD is different from impulsivity in ADHD and, whether impulsivity in BPD is merely a feature of the comorbid or underlying condition of ADHD. Research on the neuropsychological basis of the behavioral overlap is scarce and remains contradictory. Yet, two studies cited in the previous review found results indicating that impaired inhibition is a core feature in adults with ADHD but not in adults with BPD [24, 25]. These studies were starting point for research activities dealing further with the question if deficient inhibitory control characterizes BPD independently of ADHD or if both disorders share response inhibition problems as a common symptom. The recent studies published since then are presented in the following and in detail in Table 1.

**Table 1:** Overview on studies concerning impulsivity in ADHD and BPD

Author	Design	Population	Description	Result
Kulacaoglu et al. 2017 [26]	Questionnaire study	Adult women with BPD (*n* = 90) and control group (CG, *n* = 90)	Self-ratings of impulsivity with the BIS and of ADHD symptoms with the ASRS.	• Higher impulsivity ratings and more ADHD diagnoses in the BPD group • Correlation between ADHD symptoms and impulsivity score on BIS • Motor impulsivity (BIS) predicted ADHD symptom score
O’Malley et al. 2016 [11]	Neuropsychological laboratory study measuring attentional functioning and impulsivity in form of response inhibition	Adults with ADHD (*n* = 40) and adults with ADHD+BPD (*n* = 20)	Self and informant ratings of impulsivity with BAARS. Attention was measured with LCT and VE. Response inhibition was measured with HSCT, SNST and MFFT.	• Comorbidity: ADHD < ADHD + BPD • Intellectual functioning: ADHD > ADHD + BPD • Impulsivity (informant-rating): ADHD < ADHD+BPD • Impulsivity (self-rating): ADHD = ADHD+BPD • Attention problems: ADHD < ADHD+BPD • Response inhibition: ADHD = ADHD+BPD
Van Dijk et al. 2014 [18]	Neuropsychological laboratory study measuring response inhibition	Adults with ADHD (*n* = 14), BPD (*n* = 12), ADHD+BPD (*n* = 7) and control subjects (*n* = 37)	Measurement of response inhibition with AX-CPT.	• ADHD: slower reaction time in AY-trials • BPD: more errors, slower reaction time in BX-trials, more errors in BX and AX trials

BIS Barratt Impulsiveness Scale, ASRS Adult ADHD Self-Report Scale, BAARS Barkley Adult ADHD Rating Scale, LCT Letter Cancellation Test, VE Visual Elevator Test, HSCT Hayling Sentence Completion Test, SNST Stroop Neuropsychological Screening Test, MFFT Matching Familiar Figures Test, AX-CPT AX-Continuous Performance Task

A questionnaire study [26] using self-rating measures reported a high number of ADHD diagnoses and significantly higher impulsivity ratings and ADHD symptom load in a group of 90 female BPD patients compared to a healthy control group. The ADHD symptom score of these study participants significantly correlated with the total score on the BIS. All subscale-scores of the BIS were significantly higher in the BPD patient group compared with the control group. The motor impulsivity subscale was predictive of the ADHD score.

O’Malley et al. [11] compared a group of ADHD patients with a group of patients meeting diagnostic criteria for both, ADHD and BPD. The comorbid group had a significantly higher psychopathological symptom load and was much more impaired in psychosocial life and intellectual functioning. Informant ratings of impulsivity were higher in the comorbid group. Self-reported impulsivity levels did not differ between groups. Impairment in attention measures was more pronounced in the comorbid group but not measures of response inhibition.

A classical test for cognitive control, ADHD related attention problems and response inhibition deficits is the Continuous Performance Test (CPT) [27]. In the CPT, participants see or hear a series of stimuli and have to either give or inhibit a response depending on the task instructions. The Conners‘CPT is used to test for ADHD [28]. CPT‘s are based on the paradigm that a global prepotency of response is created that must occasionally be overridden by voluntary control. A further development of the CPT is the AX-CPT [29]. It takes into account that response inhibition also depends on context processing. Thus, this test measures a person’s ability to maintain a goal state (e.g., that X must follow A to be a target), and the ability to process context (e.g., knowing that if a B is presented, the next letter cannot be a target). The AX-CPT was used as a possible means to differentiate between ADHD and BPD in a first pilot study with 14 ADHD patients, 12 BPD patients, 7 patients with ADHD and BPD and 37 healthy controls [18]. ADHD and BPD patients both had problems with response inhibition. Patients with BPD had the highest overall error rate in comparison to the other groups. BPD patients had more problems to use context factors as hints for inhibition whereas ADHD patients had slower responses when they had to interrupt an already ongoing response tendency. The authors conclude that both patient groups show inhibition problems with BPD patients having more - and more pervasive - deficits compared with the ADHD patients. The results suggest that inhibition deficits are part of BPD independently of ADHD and that context processing is impaired in BPD.

However, directly linking cognitive tests to behavior is not always possible since associations between neuropsychological test results and symptoms or behavior are often weak and a causal link has not been clearly demonstrated. It is nevertheless interesting that impaired response inhibition in the narrow sense (interruption of ongoing actions) is reported for ADHD and not for BPD – in line with the idea that impulsivity in ADHD reflects a more neurocognitive disorder whereas impulsivity in BPD has a more “behavioral” and composite etiology.

#### Impulsivity under stress

In the framework of hypotheses generated based on scientific work related to the theories and assumptions of Marsha Linehan’s Dialectical Behavioral Therapy (DBT) stress has been assumed to be a powerful factor influencing levels of impulsivity especially in BPD patients. To understand the differences and commonalities between ADHD and BPD in the realm of impulsivity better it has been hypothesized that ADHD is characterized by high levels of trait impulsivity whereas BPD is characterized by state impulsivity or, in other words, stress-induced high impulsivity levels.

To elucidate these assumptions, Cackowski et al. [19] investigated the effect of induced stress on different components of impulsivity in a group of female BPD patients compared with matched controls. The results were controlled for the level of self-reported ADHD symptoms. BPD patients reported higher levels of trait impulsivity compared with the control group. In both groups, stress-induction lead to higher levels of self-reported impulsivity. BPD patients reported higher state impulsivity under the resting and the stress condition and a higher stress-dependent increase in state impulsivity. The neuropsychological tasks in this study revealed response inhibition deficits in a go/stop task in BPD patients under stress. Decision making was not different in women with BPD and the control group under both conditions.

With the aim to further investigate the impact of acute stress on self-reported and neuropsychological measures of impulsivity in ADHD and BPD the study from Krause-Utz et al. [21] followed this line of thought that links impulse control deficits under non-stress conditions to ADHD and assumes that impulse control deficits in BPD are primarily related to experiences of acute stress (see Table 2). The study confirmed that action withholding is stress-dependent in BPD. At the same time the study contributed to the differentiation of impulsivity features in both disorders, showing that delay discounting seems to be a feature of BPD independent of ADHD and stress.

**Table 2:** Overview on studies concerning impulsivity under stress in ADHD and BPD

Author	Design	Population	Description	Result
Krause-Utz et al. 2016 [21]	Neuropsychological laboratory study with stress induction measuring inhibitory control	Adult women with BPD (*n* = 30), adult women with ADHD (*n* = 28) and CG of women (*n* = 30)	Self-ratings of impulsivity with BIS and UPPS. Stress induction with MMST. Measurement of inhibitory control with action withholding (IMT), action cancelation (GoSTop) and delay discounting task.	• Trait impulsivity: ADHD > BPD > CG • State impulsivity after stress higher in all groups and ADHD = BPD > CG • Action withholding before stress: ADHD = BPD > HC • Action withholding deficits after stress significantly higher in BPD (not in ADHD and CG) • Action cancelation: no differences in inhibition and no stress effect • Delay discounting: BPD > CG. Preference for immediate reward in ADHD. No stress effect.
Cackowski et al. 2014 [19]	Neuropsychological laboratory study with measurement of response inhibition and decision making before and after stress induction	Adults women with BPD (*n* = 31) and CG of women (*n* = 30), ADHD score controlled for.	Self and informant ratings of impulsivity with BIS, UPPS, STAXI and STIMP. Stress-induction with MMST. Neuropsychological measurement of response inhibition with go/nogo task and decision making with IGT.	• Trait impulsivity: BPD > CG • State impulsivity before and after stress: BPD > CG • Stress-dependent increase in state impulsivity: BPD > CG • Response inhibition problems under stress: BPD (ADHD-score controlled for) > CG • Decision making before and after stress: BPD = CG

UPPS Urgency Premeditation Perseverance Sensation Seeking Impulsive Behaviour Scale, MMST Mannheim Multicomponent Stress Test, IMT Immediate Memory Task, STAXI State Trait Anger Expression Inventory, STIMP State Impulsivity Questionnaire, IGT Iowa Gambling Task

#### Impulsivity and association to early traumatic experiences

Impulsivity in different psychiatric disorders and its association with early trauma experiences is subject of a study in 744 participants published by Richard-Lepouriel and colleagues [12]. Six clinical patient groups (bipolar disorder (BD), BPD, ADHD and comorbid patients with BPD + BD, BPD + ADHD and BD + BPD + ADHD) and a control group were compared. Interestingly, impulsivity measures were the same in BD and the control group, whereas patients with BPD + ADHD reported higher levels of impulsivity. Impulsivity was associated with traumatic experiences in BD and the control group but not in BPD and ADHD.

Another study compared 165 BPD patients and 165 healthy controls in terms of impulsivity, traumatic experiences, ADHD symptoms and dissociative symptom load [17]. High scores in all the investigated measures were confirmed in the BPD group. Attentional and motor impulsiveness were predictors of ADHD in BPD patients.

#### Emotional Dysregulation

In BPD, difficulties of emotion regulation are considered the core pathology [30]. In ADHD, a considerable subgroup of patients also suffer from emotional dysregulation and some authors consider emotional dysregulation a primary symptom in ADHD [8, 31].

Rüfenacht et al. [13] compared psychometric measurements of emotion regulation abilities between four groups: 279 patients with ADHD, 70 patients with BPD and 60 patients with ADHD+BPD, and a group of controls. All clinical groups had high emotional reactivity, including emotional sensitivity, intensity and persistence of emotions. This characteristic was most pronounced in patients with comorbid ADHD+BPD, followed by BPD. But emotional reactivity was more pronounced in ADHD than in controls. ADHD patients had better control over their emotions and used more adaptive cognitive strategies and fewer non-adaptive cognitive strategies than BPD patients. ADHD and BPD patients were similarly impaired in the perception of self and others. Cognitive empathy scores were lower in the comorbid group than in the ADHD group.

Substance use disorders (SUD) are important comorbidities of ADHD and BPD patients. A mediation analysis in 305 male SUD patients confirmed that difficulties in emotion regulation did partially mediate the relationship between ADHD symptoms and symptoms of BPD [32].

In another attempt to contribute some more explanations on the relationship between retrospectively reported symptoms of ADHD in childhood and BPD, Fossati et al. [33] assessed 207 outpatients with different personality disorders. The contribution of both - symptoms of impulsivity and emotional dysregulation - as mediators on the relationship between ADHD symptoms and BPD features was confirmed in the participating women but not in men.

### Shared genetic, epigenetic and environmental risk factors for ADHD and BPD

Little is known about shared risk factors for ADHD and BPD. Overlapping genetic risks raise interesting questions about the distinctiveness of the categorial diagnoses.

#### Genetics

Heritability estimates of ADHD are ranging between 70 and 80% [34] while familial and twin studies indicate a heritability of BPD ranging from 35 to 46% [35]. Interestingly, self-reported ADHD symptoms in adults have very similar heritability in the range of 35–50%. The lower heritability estimates in BPD may thus reflect truly lower heritability or could also be related to rater effects - or be caused by not using full clinical criteria. These aspects of heritability in ADHD have been discussed by Larsson and colleagues [36].

The most robust findings in the field of genetic risk arise from estimating genetic overlap using genome wide association studies (GWAS). Polygenic risk scores (PRS) are calculated as an estimate of an individual’s genetic liability to a trait or disease, according to their genotype profile and relevant GWAS data [37].

A GWAS of BPD indicated a genetic overlap with bipolar disorder (BD), major depression and schizophrenia [16]. Genetic overlap of BPD with BD (r_g_ = 0.28 [*P* = 2.99 × 10^− 3^]), schizophrenia (r_g_ = 0.34 [*P* = 4.37 × 10^− 5^]) and major depression (r_g_ = 0.57 [*P* = 1.04 × 10^− 3^]) was found. We have not found a GWAS that directly examined the genetic overlap between ADHD and BPD.

In a prospective population-based study using a PRS-approach, Mistry et al. [15] examined associations between the genetic risk for BD and childhood ADHD as well as BPD-traits. The children included were age 7.6 when assessed for ADHD via parent-interviews and 11 years when assessed for BPD-traits (using the Childhood Interview for DSM-IV Borderline Personality Disorder). There was no association between BD-PRS and being high risk for BPD (OR = 1.01, 95% CI = 0.90, 1.13; *p* = 0.860) whereas a strong association between BD-PRS and increased odds of ADHD-diagnosis was found (OR = 1.31, 95% CI = 1.10, 1.57; *p* = 0.003). Again, a direct association between ADHD and BPD has not been examined in this study.

Kuja-Halkola et al. [38] investigated the co-occurrence and familial co-aggregation between clinically diagnosed ADHD and BPD in over two millions individuals born in Sweden between 1979 and 2001. The authors estimated the within-individual association between ADHD and BPD and the familial occurrence of ADHD and/or BPD in twins, siblings, half siblings and cousins, using an adjusted odds ratio (aOR). Individuals with an ADHD diagnosis had aOR of 19.4 of also having a BPD diagnosis, compared to individuals not diagnosed with ADHD. Having relatives with ADHD also increased the risk for BPD, with higher aORs for closer relatives and smaller aORs for more distant relatives. The pattern of familial coaggregation of ADHD and BPD across different types of relatives indicates that genetic factors play a role in the co-occurrence of ADHD and BPD. And the results also showed that not all of the association was explained by genetic factors.

A review and meta-analysis of genetic factors in BPD did not identify a susceptibility gene for the development of BPD [35]. The authors discuss the existing evidence for gene–environment correlations in the genesis of BPD. They propose more research of candidate genes that modulate vulnerability towards environmental factors, e.g. genes implicated in HPA axis regulation.

#### Epigenetics

Amad et al. [35] pronounced the importance of epigenetics in the context of gene-environment-interactions (GxE interactions). Epigenetic changes have been a topic of interest in psychiatric research as they might represent „fingerprints “of GxE interactions. An early epigenetic study revealed changes in DNA methylation in peripheral tissue in boys with ADHD compared with controls [39]. A more recent study – and the only study we found showing an association at epigenetic level – reported a link between childhood trauma, methylation of the Serotonin 3A receptor (5-HT_3A_R) and the severity of ADHD, BPD and BD [40]. Following previous studies that suggested overlapping sets of common genetic risk factors in psychiatric disorders, such as alterations in serotonin receptors that play an important role in the development of the human brain, the authors hypothesized that 5-HT_3A_R methylation status will mediate the effect of childhood maltreatment on psychopathology in adulthood. For the methylation analysis, blood samples were taken from the three clinical groups (ADHD *N* = 111, BPD *N* = 116 and BD *N* = 122). The results revealed an association between maltreatment and severity of the disorder. There was a significant association of childhood maltreatment, especially physical abuse, with 5-HT_3A_R methylation levels.

#### Adverse childhood events

The interrelation between ADHD, traumatic experiences and BPD is still subject of debate. GxE-interactions are thought to be important factors in the development of symptom-expression in both BPD and ADHD. In general, individuals with a particular genotype are at risk to develop more severe symptoms in the presence of environmental adversity of any sort [41, 42].

Brown et al. [43] investigated adverse childhood events (ACE) in children diagnosed with ADHD using data obtained from the 2011/2012 National Survey of Children’s Health, a cross-sectional telephone survey. Included were data of parents of children in the United States who responded to queries about ADHD and their child’s exposure to nine different ACEs (e.g. socioeconomic hardship, domestic violence, discrimination). The 76,227 children included were aged 4–17 years. A graded relationship between ACE score and the parent-reported severity of ADHD-symptoms was observed. Children with ACE scores of 1 (aOR, 1.60; 95% CI, 1.38–1.87), 2 (aOR, 2.16; 95% CI, 1.81–2.57), 3 (aOR, 3.09; 95% CI, 2.46–3.88), and 4 (aOR, 3.97; 95% CI, 3.29–4.80), were more likely to have parent-reported ADHD compared with children without ACEs. The authors critically mentioned the fact that only a minority of the pediatricians systematically screen for ACEs. The consequences of possible ADHD misdiagnosis due to behavioral patterns connected to underlying trauma or neglect and the general arguments about routine screening for ACEs during ADHD assessment in childhood [44] are discussed.

Dalbudak and Evren [45] investigated shared risk factors for ADHD and Borderline personality features (BPF) in a sample of 300 Turkish university students. The severity of BPF correlated with adult ADHD symptoms, emotional abuse, physical abuse and depression scores. Hierarchical regression analysis indicated that, among depressive symptoms and history of emotional or physical abuse, the severity of ADHD symptoms is a predictor for the severity of BPF. This is in line with previous findings that showed a relation between childhood symptoms of ADHD, emotional abuse in childhood and more severe BPF in adulthood [5, 22, 46].

Ferrer et al. [10] analyzed the history of childhood trauma retrospectively in adult patients with BPD, ADHD, BPD + ADHD and controls. Patients with BPD + ADHD reported more history of maltreatment during childhood, compared with healthy controls and non-comorbid BPD and ADHD patients.

For total trauma-scores, there was a highly significant gradual increase: “No BPD-no ADHD” (48.45) < ADHD (53.64) < BPD (54.99) < BPD + ADHD (61.16). The same ranking applied to total abuse, emotional abuse and sexual abuse. According to the total trauma-scores, comorbid patients seem to suffer more childhood trauma than non-comorbid patients do. The ADHD-group has slightly lower scores compared with the BPD-group.

The recent investigations on ACE and severity of ADHD add further support to the evidence already discussed in our preceding review: Not only patients with BPD but also patients with ADHD - and especially comorbid ADHD+BPD-patients - have increased rates of childhood trauma and adverse events. Having ADHD could go along with one of the genetic backgrounds on which environmental risks play - leading to development of BPD. Thus, children with ADHD might be more likely to develop BPD when exposed to trauma.

### Treatment

Treatment strategies for BPD are mostly psychotherapeutic [47, 48]. Treatment guidelines for ADHD often recommend multimodal approaches with medication combined with psychosocial treatment [49]. Even if a considerable number of BPD patients also suffer from ADHD, knowledge about treatment of comorbid patients suffering from both, ADHD and BPD, is rare so far.

#### Treatment strategies for comorbid ADHD+BPD

Asherson et al. [50] stated a shortage of data about treatment of adults with ADHD and comorbid BPD after reviewing the literature up to 2013. Based on the evidence and the authors’ clinical experience, they recommended that treatment of ADHD should *always* be considered when treating comorbid personality disorders, with the aim to reduce ADHD-dependent distress, improve functioning in the daily lives and enhance control over the behavior. According to Asherson and colleagues, patients are more likely to engage and benefit from psychological treatment programs for BPD when comorbid ADHD is treated.

Beneficial but small to moderate effects of methylphenidate (MPH) on emotional dysregulation in adult ADHD (without taking into account comorbidity) have been shown in the past [51] and confirmed in more recent meta-analyses [52, 53]. In a naturalistic study, Prada et al. [14] investigated therapy outcome of comorbid BPD-ADHD patients receiving DBT therapy with or without additional MPH treatment. BPD-ADHD patients who were receiving MPH treatment showed a significantly improved response to DBT treatment for Trait-State Anger scores, motor impulsiveness, depression severity, and ADHD severity, when compared with those without stimulant medication.

Gvirts et al. [54] administered a single dose of methylphenidate in a randomized placebo-controlled double blind design and studied the effect on cognitive measures and decision making in 22 patients with BPD. The Test of Variables of Attention, a digit-span test, and the computerized Iowa Gambling Task was completed by the participants after they had been administered either a single dose of 20–30 mg MPH (adjusted to the patient’s weight) or a placebo. The authors report an improvement of decision-making capacities following the administration of MPH depending on ADHD symptom level: Lower inattention scores were associated with a greater improvement in decision making following the administration of MPH when compared with patients with higher inattention scores. Thus, MPH improved performance in the decision-making task in patients with BPD whose symptoms of inattention were less severe. The authors conclude that MPH may improve decision making in patients with BPD and that this effect was mediated by the level of ADHD-symptoms.

## Discussion

The evidence presented in this review illustrates that research efforts since 2014 have produced some inconsistent results, some progress in differentiation of seemingly overlapping ADHD/BPD-symptomatology and some interesting ideas for future research.

As ADHD frequently co-occurs with a wide range of other neurodevelopmental and psychiatric disorders the specificity of the clinically observed close link between ADHD and BPD has to be further defined and sharpened. The aim of such better understanding of this co-occurrence can be a basis for a better understanding of systematic nosologic and etiopathogenetic connections and might have the potential to contribute to better treatment decisions and rational preventive measures. It has been suggested that the co-occurrence of ADHD and BPD is partly due to shared genetic factors. Estimated genetic correlation may be the best way to indicate genetic overlap between disorders. This was estimated at 0.59 in an older study discussed in our previous review [55]. In our current literature research, we could not find publications that are more recent on this topic. What is certain is that there are many genes of small effect involved. In other words, BPD and ADHD are both polygenic disorders with overlap of around 60% of the genetic variants involved [16, 56]. The large population analysis from Kuja-Halkola et al. [38] confirms the high co-occurrence of ADHD and BPD on a population level with a 19.4 fold increased risk to suffer from BPD when having a diagnosis of ADHD. And it confirms that ADHD and BPD co-aggregate in relatives, confirming the probable genetic basis of this co-occurrence.

It has been proposed to look further into plausible targets of GxE interactions and epigenetics. In general, research on candidate genes, single genes and GxE findings have to be seen with caution because results have often not been replicated. As stated in our genetics-section, the most robust findings arise from GWAS-data.

We found only one study on epigenetic alterations and their association with traumatic experiences in BPD, ADHD and BD [40]. Because of the high plausibility of epigenetic changes related to traumatic stress and other environmental risk factors often referred to as invalidating environment this line of thought has to be followed in future research.

Research on environmental risk factors revealed a correlation between ADHD and adverse childhood events. Retrospectively, patients with BPD + ADHD report more childhood maltreatment. Risky, impulsive and novelty seeking behaviors in children with ADHD might elevate the risk for exposure to traumatic situations. Some authors point to the risk of misdiagnosing ADHD in children that suffer mainly from emotional neglect and abuse and therefore recommend general screening for adverse events and traumatic experiences in all children presenting for ADHD diagnosis. In doing so, impulsivity features that can be correlated to traumatic experiences might be clearly distinguishable from ADHD, leading to fewer misdiagnoses. Conversely, it could be argued, that experiencing a trauma may lead to more severe ADHD symptoms. Thus, the precise nature of the interrelation between ADHD, traumatic experiences, impulsivity and BPD is still subject of debate.

In order to understand the discussion about the genetic background of ADHD, BPD and the comorbid ADHD+BPD-patients, it is important to be familiar with the concept of pleiotropy. Genetic pleiotropy means that the same genetic background can lead to different phenotypes. In a recent review on this topic, Lee et al. [57] summarize that “It is now clear that a substantial fraction of genetic influences on psychopathology transcend clinical diagnostic boundaries.” Different underlying mechanisms are currently the subject of interest, among them genetic effects on neurodevelopment, diverse actions of regulatory elements, mediated effects.

**Fig. 1:**
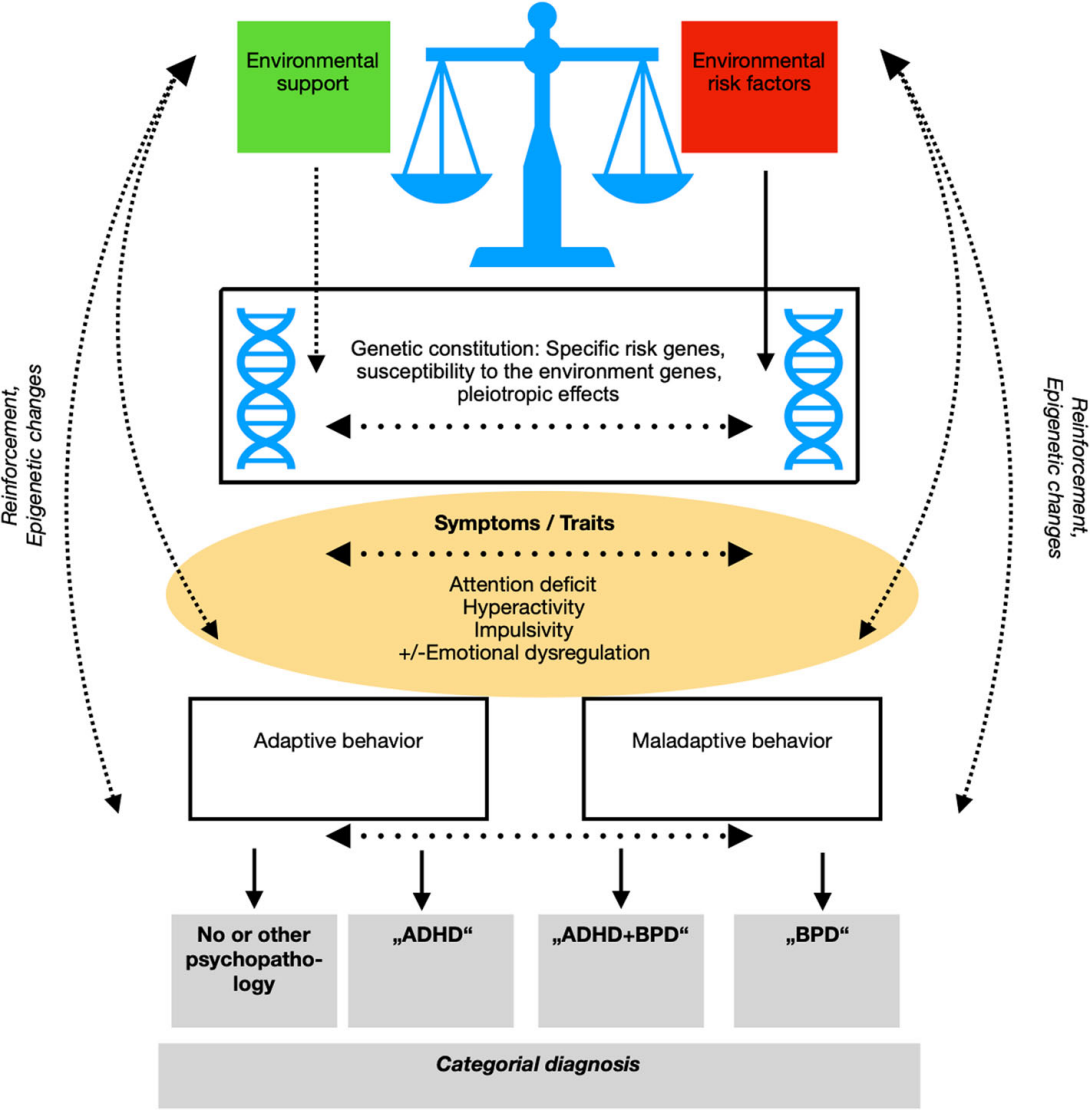
Hypothetical pathogenesis model inspired by Amad et al. [35]. The interplay of environmental factors and the genetic constitution leads to traits (e.g. impulsivity, emotional dysregulation or attention problems). These provoke reactions (supportive or invalidating). In case of adverse events (e.g. parental aggression), a mutual reinforcement (e.g. stress <− > impulsivity) +/− epigenetic effects intensify the symptoms. To reduce stress, individuals develop maladaptive behaviors that are again reinforced and repeated in the case of a predominantly negative environment. Overlapping risk factors, symptoms and behaviors lead to rather imprecise categorial diagnoses

Different potential models can be derived from the genetic, epigenetic and environmental risk factor research at this point: (1) Same genes lead to two distinct disorders (genetic pleiotropy); (2) BPD and ADHD are overlapping, e.g. they are different presentations of the same underlying disorder; (3) ADHD is leading to higher risk for developing BPD. Model [3] is another version of the pleiotropic model where having ADHD is a risk of developing BPD (a) in the presence of environmental stressors and (b) at the same time is in itself a risk factor for environmental and psychological stress (e. g. increases the likelihood of traumatic experiences). If model 3 a) applies, genetic risk factors for ADHD might as well reflect a group of susceptibility genes for BPD. Far more research is needed to clarify the causal role of stress on ADHD and on its course. As an illustration for these considerations an interesting older study by Harold et al. [58] shall be reminded of here: it investigated the influence of genetic and environmental factors and their interrelatedness with a cross sectional and longitudinal adoption-design. ADHD symptoms of the biological mother, rearing mother and the child, maternal hostility and child aggression were assessed. The biological mother’s ADHD symptoms predicted child impulsivity/activation at age 4,5, which in turn predicted maternal hostility from the adoptive mother, and later child ADHD symptoms at 6 years of age. It could be assumed that ADHD features are a risk factor for a stressful environment, starting a vicious circle (Fig. 1). If this was true, parental coaching and psychotherapy of parents with ADHD might be a key intervention to prevent comorbid personality disorders in children with ADHD. This clinically relevant topic should be further investigated. The ADHD-genotype might thus represent one of the possible genetic backgrounds on which environmental risks as for example traumatic experiences or adversities play - leading to the development of BPD.

In our previous review we postulated that ADHD children are at elevated risk for adversities and traumatic experiences in childhood and that this contributes to the development of BPD in adolescence and adulthood. Recent studies in the field of epigenetics and genetics of vulnerability-genes add interesting aspects to our previous discussion about a “sensitive” genotype in combination with a non-fitting environment in childhood. The vulnerability might not (exclusively) be dependent on disorder-specific susceptibility-genes (e.g. ADHD-related susceptibility genes) but also on epigenetic changes caused by interpersonal stress (Fig. 1). Due to the lack of evidence, this model is currently of hypothetic nature.

To decide whether BPD and ADHD are distinct or overlapping disorders, it would be helpful to know whether symptoms like impulsivity and emotional dysregulation are the same in both disorders or if there are differences in etiology and thus different treatment options. There have been several efforts to disentangle “ADHD-typical” and “BPD-typical” impulsivity and features of impulsivity and emotional dysregulation in ADHD and BPD. This line of research has seen the most differentiated progress in recent years, although most reviewed studies have small sample sizes and need confirmation through reproduction. Summarized findings of the reviewed studies on impulsivity and impulsivity under stress indicate that – quantitatively – impulsivity is a feature of both, ADHD and BPD. In addition, impulsivity level seems highest in patients with comorbid ADHD and BPD (Tables 1 and 2). Thus, impulsivity seems to be a feature of BPD independent of ADHD and not – as assumed previously – merely a symptom of an underlying ADHD. Stress-dependence of impulsivity has been found in BPD but not in ADHD. Response inhibition deficits seem to characterize both disorders. Individuals with BPD have more - and more pervasive - deficits in response-inhibition with pronounced difficulties in using context cues [18]. Individuals with ADHD in contrast have more difficulties to interrupt an already ongoing response. This indicates qualitative differences of impulsivity in ADHD and BPD, as also indicated in the descriptive diagnostic criteria. More precise distinction of impulsivity-types in ADHD and BPD is necessary and first results point to subtle differences: Whereas motor impulsivity and a failure to interrupt ongoing responses are features of ADHD, BPD patients present an inability to use context information to inhibit prepotent response tendencies. The presented neuropsychological differentiations seem to point to a proximity of BPD and schizophrenia spectrum disorders in terms of impulsivity.

Emotion regulation is a symptom of both disorders and patients with ADHD, patients with BPD and patients with both, ADHD+BPD, seem to have – in this order – growing intensity of emotion regulation problems with the comorbid patients having the most severe emotion regulation difficulties and most dysfunctional emotion regulation habits. Emotion regulation difficulties seem to function as a mediator between ADHD symptoms and BPD features. In analogy to the discussion about impulsivity and the distinguishing features of impulsivity in ADHD and BPD, it would be interesting to further clarify the qualitative nature of emotional dysregulation in ADHD and BPD.

Treatment decisions for comorbid patients with ADHD and BPD today are based on expert advice and not on systematic scientific evidence. Only one naturalistic study reported on methylphenidate treatment in patients with BPD and ADHD passing through a DBT-treatment program reported promising results.

The above-mentioned conceptualization of the relationship between ADHD and BPD might also have implications for future ideas about treatment options. Currently there are no known predictors of good or poor response to ADHD drug treatments. Trauma has not been identified as a predictor of poor response to drug treatments in ADHD - this might be an important area for future enquiry. A background of trauma may or may not be associated with a good response to medications. To date, this discussion remains speculative, as causal directions are not well investigated.

Based on the presented evidence and models it is conceivable that BPD may be a heterogeneous entity that develops based on unfavorable learning histories or traumatic experiences in the context of early otherness (e. g. developmental disorders) and in the absence of a fitting environment or in the context of adverse events or childhood trauma. Some authors have discussed subtypes of BPD and have considered that severe ADHD with associated behavioral and emotional symptoms might reflect a subtype of BPD. Other factors that have important implications for the treatment decisions are other developmental disorders, organic disorders with psychiatric symptoms and complex PTSD that lead to similar behavioral and emotional symptoms. Another possibility is that ADHD and BPD are different entities and co-occur in individuals.

## Conclusion

Knowledge about the relationship between ADHD and BPD has grown since 2014. Results of genetic studies remain unspecific and inconsistent. Epigenetic research and research focussing on hypothesized vulnerability genes or sites seem a promising avenue in the future (see Fig. 1). Progress in the differentiation of similar symptomatology, namely in the symptom domains of impulsivity and emotional dysregulation, has been made and future treatment ideas should take into account the differences in impulse control in ADHD and BPD. Evidence on treatment of the comorbid condition remains scarce and future research is needed to allow rational treatment decisions. The lack of studies dealing with treatment of the comorbid condition indicates the necessity of further research. Studies on transdiagnostic treatment options for symptom domains such as impulsivity and emotional dysregulation might be helpful to clarify the question whether these are of the same kind or in both disorders or fundamentally different. In general it can be stated that research on the comorbidity and overlap of ADHD and BPD is still limited with often too small sample sizes and variable methodology, leading to inconsistencies.

## Data Availability

Data sharing not applicable to this article as no datasets were generated or analyzed during the current study. Alexandra Philipsen: AP declares that she served on advisory boards, gave lectures, performed phase 3 studies, or received travel grants within the last 5 years from Eli Lilly and Co, Janssen-Cilag, Lundbeck, MEDICE Arzneimittel, Pütter GmbH and Co KG, Novartis, Servier, and Shire; and has authored books and articles on ADHD published by Elsevier, Hogrefe, Schattauer, Kohlhammer, Karger, Oxford Press, and Springer. Swantje Matthies: No conflicts of interest.

## Abbreviations

ACE: adverse childhood events
ADHD: attention deficit hyperactivity disorder
AX-CPT: AX- Continuous Performance Test
BD: bipolar disorder
BIS: Barratt Impulsiveness Scale
BPD: borderline personality disorder
BPD-ADHD: comorbid attention deficit hyperactivity disorder and borderline personality disorder
CPT: Continuous Performance Test
HPA-Axis: hypothalamic–pituitary–adrenal axis
GxE interactions: gene-environment-interactions
GWAS: genome wide association study
MPH: methylphenidate
OR: odds ratio
aOR: adjusted odds ratio
SUD: substance use disorder
PRS: polygenetic risk score
DSM-V: diagnostic and statistical manual of mental disorders
